# Giant lipoma arising from deep lobe of the parotid gland

**DOI:** 10.1186/1477-7819-4-28

**Published:** 2006-06-02

**Authors:** Che-Wei Wu, Hung-Pin Chi, Feng-Yu Chiang, Ying-Che Hsu, Leong-Perng Chan, Wen-Rei Kuo

**Affiliations:** 1 Department of Otolaryngology – Head and Neck Surgery, Kaohsiung Medical University Hospital, Taiwan; 2 Department of Otolaryngology – Head and Neck Surgery, Faculty of Medicine, College of Medicine, Kaohsiung Medical University, Taiwan

## Abstract

**Background:**

Lipomas are common benign soft tissue neoplasms but they are found very rarely in the deep lobe of parotid gland. Surgical intervention in these tumors is challenging because of the proximity of the facial nerve, and thus knowledge of the anatomy and meticulous surgical technique are essential.

**Case presentation:**

A 71-year-old female presented with a large asymptomatic mass, which had occupied the left facial area for over the past fifteen years, and she requested surgical excision for a cosmetically better facial appearance. The computed tomography (CT) scan showed a well-defined giant lipoma arising from the left deep parotid gland. The lipoma was successfully enucleated after full exposure and mobilization of the overlying facial nerve branches. The surgical specimen measured 9 × 6 cm in size, and histopathology revealed fibrolipoma. The patient experienced an uneventful recovery, with a satisfying facial contour and intact facial nerve function.

**Conclusion:**

Giant lipomas involving the deep parotid lobe are extremely rare. The high-resolution CT scan provides an accurate and cost-effective preoperative investigative method. Surgical management of deep lobe lipoma should be performed by experienced surgeons due to the need for meticulous dissection of the facial nerve branches. Superficial parotidectomy before deep lobe lipoma removal may be unnecessary in selected cases because preservation of the superficial lobe may contribute to a better aesthetic and functional result.

## Background

Lipomas are the most commonly encountered benign mesenchymal tumors, arising in any location where fat is normally present. Their occurrence in the head and neck is relatively rare [1], most commonly presenting in the posterior subcutaneous neck [2,3]. However, lipomas involving the deep parotid lobe are extremely rare. The first case of lipoma involving the deep lobe of parotid gland was reported by Janecka *et al*., in 1977 [4], and few additional cases of deep lobe parotid lipomas have been reported in the surgical literature since then [2,5-9], Surgical intervention in these tumors is challenging because of the proximity of the facial nerve, and thus knowledge of the anatomy and meticulous surgical technique are essential. Here, we describe a rare case of giant lipoma arising from deep lobe of the parotid gland that was successfully managed by surgery.

## Case presentation

**Figure 1:**
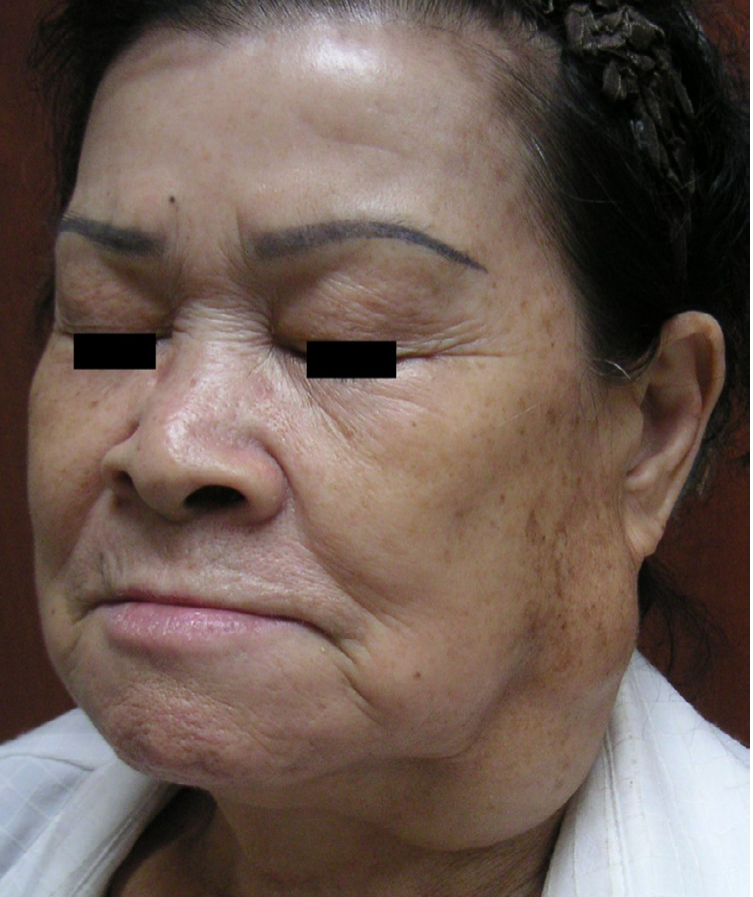
Photograph of a 71-year-old female patient who presented with a 15-year history of slow-growing swelling of the left parotid and upper lateral neck area.

A 71-year-old female presented in our clinic with a large left facial mass requesting surgical excision for a cosmetically better facial appearance. She had been aware of the slow-growing, painless swelling for over the past fifteen years. She had sought medical advice ten years before, but she had been unwilling to undergo surgery due to the fear of the postoperative morbidity. Clinical examination revealed a mobile, soft, and non-tender mass measuring 10 × 7 cm in size over the region of the left parotid and upper lateral neck area (Figure 1). The surface of the mass was smooth and the overlying skin was normal without any signs of discoloration or tumor-adhesion. The facial nerve function was intact and the remainder of the head and neck examination was unremarkable.

**Figure 2:**
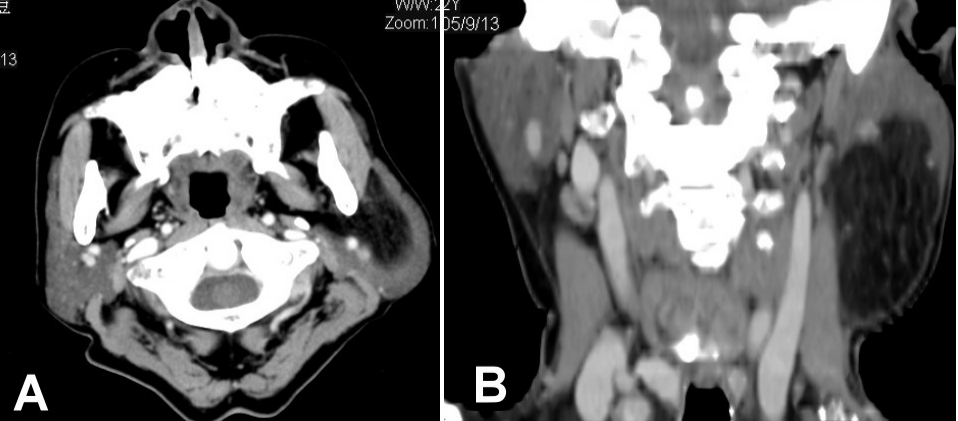
High-resolution post-contrast CT scans shows a spherical, well marginated, lower attenuation giant lipoma arising from left deep parotid lobe. (A) Axial view, the lipoma with rim of parotid gland overlaying lateral margin. (B) Reformatted coronal view, the lipoma is clearly defined from adjacent structures.

**Figure 3:**
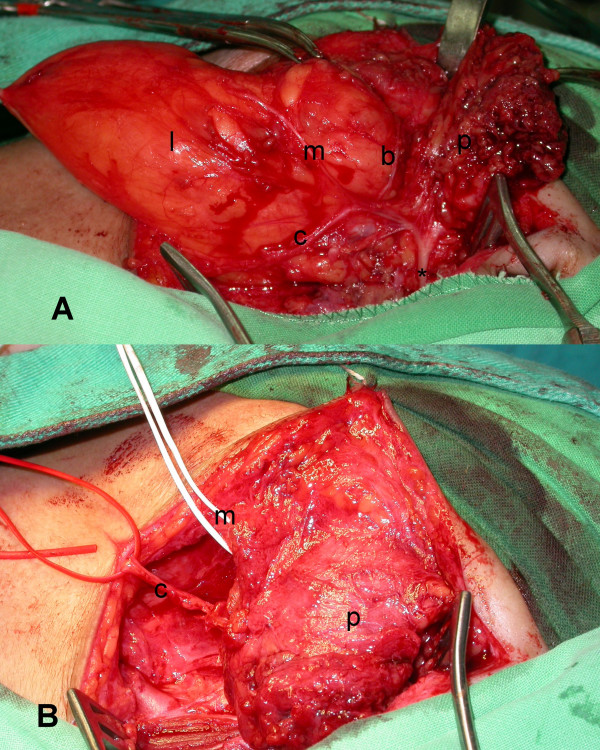
(A) The skin flaps and superficial parotid lobe (p) have been raised and a giant lipoma (l) seated on deep parotid lobe is clearly seen under the cervical (c), mandibular (m) and buccal(b) branches of the facial nerve, which were dissected from the main trunk (*). (B) Reposition of the raised superficial parotid lobe (p) after tumor removal.

**Figure 4:**
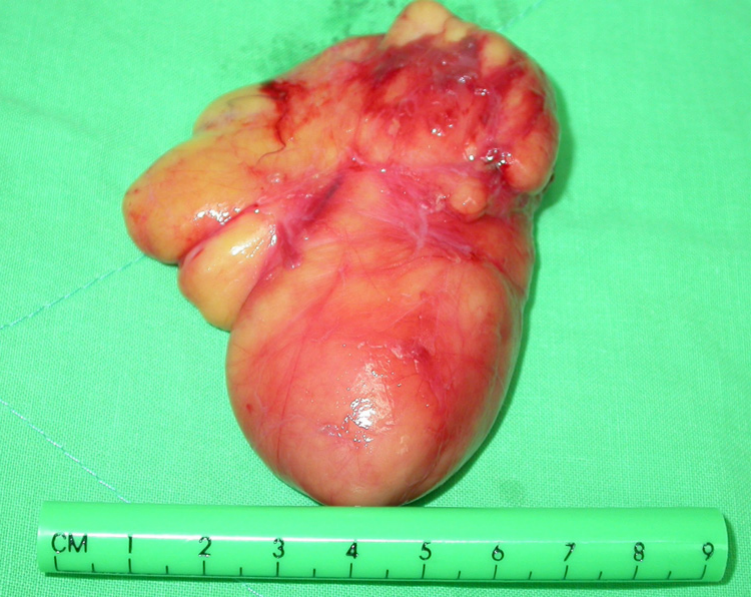
The resected well encapsulated lipoma measured 6 × 9 cm in size.

**Figure 5:**
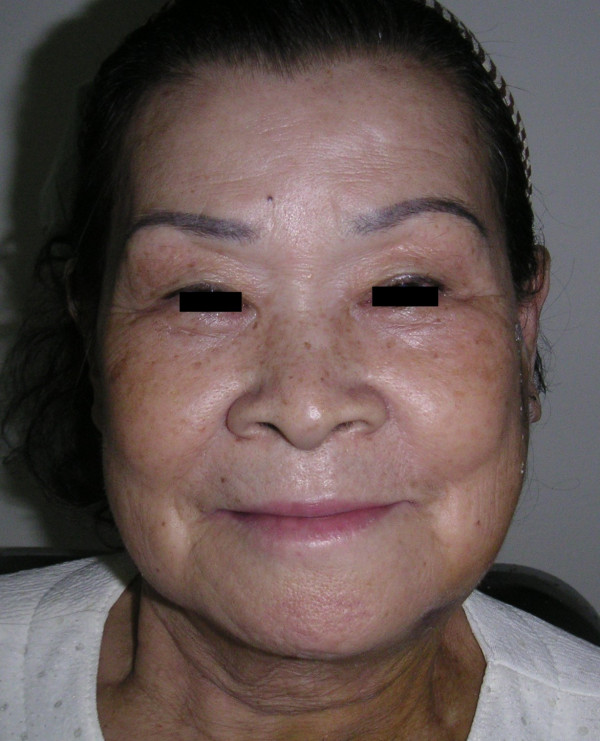
Postoperative photograph of the patient with a satisfying facial contour and intact facial nerve function.

The high-resolution CT scan showed an adipose tissue signal density mass arising from the left deep-lobe parotid gland that protruded inferiorly to the upper lateral neck (Figures 2A, B). A classic parotidectomy incision was made and the main trunk of facial nerve identified at the stylomastoid foramen. From the main trunk of the facial nerve, each branch in continuity is dissected away from the overlying superficial parotid lobe. Then a glistening yellow mass was identified in the deep lobe, closely associated with the lower branches of the facial nerve (Figure 3A). The tumor was well encapsulated and easily and totally enucleated after mobilization of the overlying facial nerve branches. Then the raised superficial parotid lobe was repositioned to cover the facial nerves before wound closure (Figure 3B). The surgical specimen was well encapsulated measuring 9 × 6 cm (Figure 4). The histopathology examination revealed fibrolipoma, a histological variant of lipoma. The patient had an uneventful recovery, with a satisfying facial contour and intact facial nerve function (assessed as House-Brackmann Grade 1) (Figure 5). Neither tumor recurrence nor Frey's syndrome was observed 9 months after surgery.

## Discussion

Lipomas are the most commonly encountered benign mesenchymal tumors that are histologically similar to mature adipose tissue, but the presence of a fibrous capsule helps to differentiate them from simple fat aggregations [10]. Onlyapproximately 25% of lipomas and their variants arise in the head and neck region [1] and most of these occursubcutaneously in the posterior neck [2,3]. Rarely, they can develop in the parotid gland with reported incidence ranges from 0.6 to 4.4% among parotid tumors, and they appear most frequently in the fifth and sixth decades of life with a definite male predominance [11].

Lipomas involving the deep parotid lobe are extremely rare. Similar to lipomas in other part of body, they tend to grow insidiously and give rise to few symptoms other than the effect of a localized mass or cosmetic concerns [9]. The same was true of this case, who had been aware of the slow-growing, painless swelling for the past fifteen years and sought medical advice only on cosmetic grounds.

As they grow, deep lobe parotid lipomas tend to extend into adjacent loose connective tissues of the neck with various shapes. They may extend posteromedially between the sternocleidomastoid and digastric muscles causing an asymptomatic soft lump on the upper lateral neck, as presented in this case. In addition, they may also extend medially into the parapharyngeal space, causing medial displacement of the lateral pharyngeal wall and/or tonsil[12]. Facial paralysis [13] and pain [11] in the presence of parotid lipoma are uncommon and have been described rarely. Probably because of the slow and flexible growth pattern of the tumor, there was neither facial paralysis nor pain on presentation even though severe tenting of the facial nerve branches was observed during operation in this case.

Clinical examination alone is insufficient to identify the nature and location of deep parotid lipomas. Hence, imaging examination such as ultrasonography, CT or magnetic resonance imaging (MRI) may be helpful in further assessment and diagnosis. Ultrasonography has been used as an initial imaging study in cases suspected to have head and neck lipomas [14,15]. Compared with CT and MRI, ultrasonography is quick, easy, less costly, and with the use of high-frequency transducers, more suitable for imaging superficial structures. However, the soft tissue characterization is less specific with ultrasonography than with CT or MRI.

On CT scans, lipomas have the typical characteristics of homogeneous masses with few septations, a specific range of CT Hounsfield Unit (HU) values (usually between -50 and -150 HU), and they show no contrast enhancement [5,15,16]. MRI can also accurately diagnose lipomas preoperatively by comparison of signal intensity on T1-and T2-weighted images [5,16]. Moreover, the margin of a lipoma is clearly defined by MRI as a 'black-rim', enabling lipomas to be distinguished from surrounding adipose tissue, a distinction that cannot be made from CT images [8].

In this reported case, however, the high-resolution CT scan provided enough information with respect to the preoperative planning and contributed to the diagnosis. Although MRI may prove to be a better diagnostic tool regarding tumor margin characteristics, this did not change our surgical strategy and it was not necessary to modify the operation based on the MRI differing from CT findings. In addition, the cost of MRI is nearly three times that of CT and so we believe that although MRI is highly useful, the CT scan with specific radiodensity recording is the preferred preoperative investigation.

Fine needle aspiration biopsy (FNAB) requires an experienced cytologist, but it still has a significant false negative rate in salivary gland tumors. It has also proved to be unreliable in diagnosing parotid lipomas [8,9,17]. Furthermore, fibrosis or adhesion between the facial nerve branches and the lipoma capsule following FNAB may be encountered, and this may increase the risk of facial nerve injury during surgery. Therefore, we did not perform FNAB for preoperative cytological diagnosis of parotid lipoma in this case.

Conservative follow-up might be a valid option for patients with clinically static deep parotid lipomas, since lipomas can now be confidently recognized by CT and MRI. Surgical intervention in these tumors is challenging and may be reserved for patients with cosmetic or pressure effects. Possible postoperative morbidities, such as facial nerve dysfunction, facial scar or asymmetric contour, and Frey's syndrome must be explained to the patient before operation. During surgery, most surgeons recommend superficial parotidectomy with facial nerve dissection before removal of the deep lobe parotid lesions [6-9,12]. Transient or temporal facial nerve dysfunction may be encountered after surgery in the case of deep lobe parotid lipoma, so full exposure of the facial nerve [9] and also facial monitoring [8,9] are advised for prevention of that morbidity.

In our surgical experience of this case, because the lipoma was mainly associated with the lower branches of the facial nerve, we exposed these branches from the main trunk by meticulously dissecting its overlaying superficial parotid lobe instead of performing a formal superficial parotidectomy. Then we reposited the raised superficial parotid lobe to its original site after tumor removal. We find the procedure with preservation of the superficial parotid lobe may have the following advantages: it helps to maintain better facial contour, and the need of resection of the redundant skin can be avoided. In addition, the incidence of postoperative Frey's syndrome is reduced.

## Conclusion

Giant lipomas involving the deep parotid lobe are extremely rare. Although MRI may provide better tumor margin characteristics, the CT scan with specific radiodensity recording is the preferred preoperative investigation. Surgical management of deep lobe lipoma is challenging and should be performed by experienced surgeons due to the need for meticulous dissection of the facial nerve branches. The postoperative aesthetic and functional results should be the major concerns. Therefore, a formal superficial parotidectomy may be unnecessary in selected cases, especially when the tumor only involves the lower branches of the facial nerve. Reposition and preservation of the raised superficial parotid lobe may contribute to a better postoperative aesthetic and functional result.

## Competing interests

The author(s) declare that they have no competing interests.

## Authors' contributions

**CWW**, **HPC**: Took part in the care of the patient, organised clinical photographs and wrote first draft of the manuscript. **FYC**: study conception and design. **YCH**: Performed literature search, retrieved articles and helped with manuscript preparation. **LPC**: Examined surgical specimen, took the photograph of the specimen and helped with manuscript preparation. **WRK**: Critical revision and supervision.

All authors read and approved the final manuscript.

## Funding source

None
